# The Effect of Area Density of Polysilicon Thermocouples on Thermoelectric Performance

**DOI:** 10.3390/s25041098

**Published:** 2025-02-12

**Authors:** Shih-Ming Yang, Zen-Wen Lai, Ai-Lin Liu

**Affiliations:** Department of Aeronautics and Astronautics, National Cheng Kung University, Tainan City 701, Taiwan; n18131053@gs.ncku.edu.tw (Z.-W.L.); f04119025@gs.ncku.edu.tw (A.-L.L.)

**Keywords:** thermoelectric energy generator, CMOS process, polysilicon thermocouple

## Abstract

Thermoelectric energy generators (TEGs) that can convert body heat into electricity are considered most promising to drive wearable devices. Many TEG designs with a polysilicon thermocouple have been proposed for implementation in high-yield semi-conductor foundry services. This study shows that the area density, defined by the number of thermocouples per mm^2^, is a better index than the fill factor in evaluating TEG performance. The effects of thermocouple length, width, and spacing (between the adjacent thermocouples) on area density, and hence on TEG performance, are analyzed. For a TEG with 33 × 1 μm (length × width) co-planar thermocouples (P- and N-thermoleg side by side) and 1 μm spacing between two adjacent thermocouples, the area density is 4902 thermocouples per mm^2^ and it can deliver a 0.110 μW/cm^2^K^2^ power factor and a 12.906 V/cm^2^K voltage factor. The performance can be improved further by 57 × 1 μm stacked thermocouples (P-thermoleg above N-thermoleg) with a higher area density 8621 to achieve results of 0.110 μW/cm^2^K^2^ and 22.638 V/cm^2^K. Such a high area density not only increases TEG performance, but also improves the DC–DC converter efficiency. A 5 × 5 mm^2^ TEG chip with co-planar or stacked thermocouples is shown to deliver above 3 μW and over 3 V when operating at a 10 °C temperature difference.

## 1. Introduction

The typical daily activities of an adult can release sufficient energy in the form of body heat and motion to generate electrical power. At the advent of modern integrated circuits towards low voltage and minimal power consumption, harvesting such heat may provide a consistent and uninterrupted energy source for wearable devices. A thermoelectric energy generator (TEG) has therefore been considered promising to power wearable devices. Many TEGs with thermocouples in V–VI compounds have commonly been produced by electrochemical deposition [1], where bismuth-telluride (Bi_2_Te_3_) materials are often used because of their better thermoelectric efficiency, where TEGs are capable of delivering 2.8 mW at a 10 °C temperature difference [2]. Reviews of the figure of merit (ZT) of thermoelectric materials [3] and of Bi_2_Te_3_-based materials [4] at room temperatures have been summarized recent TEG development. However, the Bi_2_Te_3_ TEGs suffer from issues with material toxicity, availability, and miniaturization; moreover, the deposition process is often time consuming in production. Development of novel thermoelectric materials [3], low-dimensional materials [5], and semiconductor process technology [6] have been opening new avenues for TEG design.

The major challenge in wearable device applications of a TEG is its low power output (~nW) because of the small device size (~cm^2^) and low temperature gradient (<10 °C) in the thermocouples [7,8]. One way to circumvent this challenge is by following the silicon path of thin-film technology and taking the advantage of design flexibility in semiconductor foundry services. Yang et al. [9] were the first to develop a TEG with co-planar thermocouples by a standard CMOS (Complementary Metal-Oxide-Semiconductor) process, where the complementary and symmetrical pairs of P- and N-*FETs* (field-effect transistors) are readily for thermocouples. Many TEG designs have therefore adopted the thin-film layers for the high density of thermocouples on a wafer [10]. However, semiconductor TEG design has to cross three major hurdles: (1) the need of thermal isolation to retain the thermal gradient within a thermocouple, (2) the need of thermocouple material(s) to achieve the maximum energy conversion, and (3) the need of the “correct” thermocouple size for sufficiently large number of thermocouples while avoiding Joule heating.

In all TEG designs, the thermocouples are connected electrically in series and thermally in parallel. The adjacent thermocouples are also insulated to prevent heat loss and electrically isolated to avoid short-circuiting. Intuitively, the higher area density of a large number of thermocouples would surely increase TEG power and voltage output. Recent studies have also confirmed that a higher area density by having more polysilicon thermocouples [11], polysilicon germanium thermocouples [12], or metal thermocouples [13] on the same TEG footprint is beneficial to producing higher voltage factor. The so-called “fill factor”, defined by the area of active thermocouples over the area of a TEG, had previously been adopted to gauge the performance [14,15,16,17,18,19]. It was shown, as predicted, that the TEGs with a high fill factor have a higher power factor [14]. The layout of thermocouples to increase the fill factor from 25% to 91% in a TEG could improve the power and voltage [15,16], but a contradicting result was drawn by the study of a TEG with 72 thermocouples, where the best power factor was at a 15.1% fill factor rather than at 27.2% [17]. Such a conclusion is controversial and against common sense. The cost efficiency [18] and cost effectiveness [19] related to the fill factor of TEGs were also studied, but cost may never be an issue should a TEG fail to deliver sufficient power and voltage.

This study aims to investigate the effects of the thermocouple area density on semiconductor TEG performance. It will be shown that the area density, defined by the number of thermocouples per ${\mathrm{mm}}^{2}$, is a better index than the fill factor in evaluating performance. By packing more thermocouples of optimal size, one can have a higher thermocouple thermal resistance and voltage factor. Both are critical to impedance matching and voltage regulation in TEG operation.

## 2. Thermocouples for Optimal Performance

**Figure 1:**
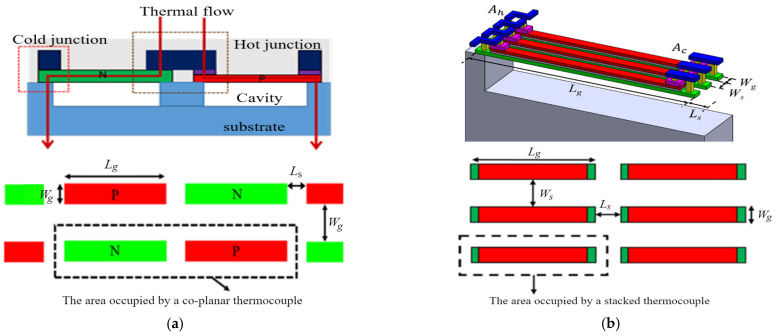
TEG configuration. $L_{g}$ and $W_{g}$ are the length/width of P- and N-thermoleg, and $W_{s}$ is the space between two adjacent thermocouples. $A_{h}$ and $A_{c}$ is the area of metal hot and cold junctions, respectively. (**a**) The co-planar thermocouple design with P- and N-thermoleg side-by-side and (**b**) the stacked thermocouple design with the P-thermoleg placed above the N-thermoleg.

Most TEGs in a hybrid configuration [9], as depicted in Figure 1a, have been known to have a sufficient thermocouple length $L_{g}$ with thermal isolation cavities above and/or below the thermocouples. TEG performance is characterized by its figure of merit, ZT:

(1) $${\mathrm{ZT}=\sigma S}^{2}T/k$$

which is proportional to the operating temperature, electrical conductivity, and Seebeck coefficient, but inversely proportional to thermal conductivity. Here, T is the temperature gradient at absolute temperature; σ and k are the electrical and thermal conductivities, respectively; and S is the Seebeck coefficient. Material with a high ZT is desirable; however, it is difficult to increase the ZT because a change in the electrical conductivity adversely affects the thermal conductivity. Therefore, the limiting factor of a semiconductor TEG is not necessarily the small Seebeck coefficient or low ZT, but rather it is the poor temperature gradient from insufficient thermal isolation, the mismatching thermal/electrical resistance of thermocouples, and an insufficient number of thermocouples (low area density). The focus of semiconductor TEG design is exploiting processing technology to achieve a high area density for higher output power and voltage.

On a wearable TEG, the extrinsic temperature difference between the body core and the ambient temperature leads to a heat flow, and the intrinsic temperature gradient across the thermocouple hot/cold junctions produces an open-circuit voltage, $V_{0}$, by the Seebeck effect:

(2) $$V_{0}=N_{g}S_{g}\Delta T_{g}$$

where $N_{g}$ is the number of thermocouples; $S_{g}$ is the Seebeck coefficient of the thermocouple; $S_{g}=S_{p}{-S}_{n}$, with the subscript for P- and N-thermolegs, respectively; $\Delta T_{g}$ is the temperature gradient between the hot/cold junctions. The power factor is defined by the power generated per unit area, per temperature difference square, and the voltage factor is defined by the voltage generated per unit area, per temperature difference. These have been employed to evaluate TEG performance [9]:

(3a) $$\phi_{P}=P_{\mathrm{out}}/\left(A_{g}\Delta T^{2}\right)$$

(3b) $$\phi_{V}=V_{0}/\left(A_{g}\Delta T\right)$$

where $P_{\mathrm{out}}={V_{0}}^{2}/\left({4R}_{g}\right)$ is the output power; $R_{g}$ is the electrical resistance of the thermocouples; $A_{g}$ is the footprint of the thermocouple, also counting the thermal insulation and electrical isolation spacing $L_{s}$ and $W_{s}$ in length and width directions, as illustrated in Figure 1a. The isolation spacings between two adjacent thermocouples are key parameter(s) to increase the area density.

In addition to the TEG configuration shown in Figure 1a, the thermocouple geometry has to be selected for matching thermal/electrical resistance for sufficient $\Delta T_{g}$. Note that the temperature gradient across the hot/cold junction may be drastically smaller than the temperature difference across the hot/cold side. Many previous semiconductor TEGs with poor output power (~nW/cm^2^K^2^) and insufficient voltage (~mV/cm^2^K) in [10] were the result of the low area density of the thermocouple and an incorrect thermocouple size. The prerequisite of a high area density is to determine the thermocouple geometry to match its thermal/electrical resistance. In practice, successful heat harvesting from the human body relies on maximizing $\Delta T_{g}$, which is challenging due to thermal resistance mismatch between the thermocouples and the TEG’s hot/cold side. The number of co-planar thermocouples, $N_{g}$, as shown in Figure 1a, is defined by

(4) $$N_{g}=A/\left(\left(L_{g}{+L}_{s}\right)\left(W_{g}{+W}_{s}\right)\right)$$

where A is the total area of the TEG chip, and $L_{g}$, $L_{s}$, $W_{g}$, and $W_{s}$ are the length of the thermoleg, length of spacing, width of the thermoleg, and width of spacing.

Based on the 1D thermal circuit model [9], the thermal resistance $\left(R_{t}\right)$ and the electrical resistance $\left(R_{e}\right)$ of a thermocouple is determined by

(5a) $$R_{t}=\left(\frac{L_{g}}{k_{p}W_{g}t_{p}}+\frac{L_{g}}{k_{n}W_{g}t_{n}}\right)/N_{g}$$

(5b) $$R_{e}=N_{g}\left(\rho_{p}\frac{L_{g}}{W_{g}t_{p}}{+\rho }_{n}\frac{L_{g}}{W_{g}t_{n}}\right)$$

where L, W, t, R, k, and ρ are the length, width, thickness, thermal resistivity, thermal conductivity, and electrical resistivity of the P- and N-thermoleg, respectively. The indices p and n refer to the P- and N-thermoleg, respectively. The thermal resistance of the hot/cold sides, $R_{h}$ and $R_{c}$, during the energy harvesting are

(6a) $$R_{h}=\frac{1}{{2N}_{g}}\frac{t_{h}}{k_{h}A_{h}}{+R}_{\mathrm{int}}$$

(6b) $$R_{c}=\frac{1}{{2N}_{g}}\frac{t_{c}}{k_{c}A_{c}}{+R}_{\mathrm{int}}$$

where R_int_ is the thermal resistance of the interface; $A_{h}$ and $A_{c}$ are the area of hot/cold junctions. The indices h and c refer to the hot and cold sides, respectively. Analysis of TEG performance is an “exact” science. An artificial neural network can be used to search for the thermocouple dimension towards optimal TEG performance [20], but this is for reference only. The 1D model has previously been developed to derive the temperature gradient across the thermocouples [9]:

(7a) $${\Delta T}_{g}=\frac{R_{e}}{N_{g}^{2}S_{g}^{2}}\left[\frac{1}{R_{c}}-\frac{1}{R_{h}}{+2C}_{2}\;\cos\left(\frac{1}{3}C_{2}\;\cos^{-1}\left(\frac{C_{1}}{C_{2}^{3}}\right)+\frac{4\pi }{3}\right)\right]$$

With

(7b) $$C_{1}=\left(\frac{1}{R_{c}}-\frac{1}{R_{h}}\right)\left(\frac{1}{R_{c}^{2}}+\frac{2}{R_{c}R_{h}}+\frac{1}{R_{h}^{2}}+\frac{1}{R_{c}^{2}}+\frac{4}{R_{t}R_{h}}+\frac{4}{R_{t}R_{c}}\right)\;+2\frac{N_{g}^{2}S_{g}^{2}}{R_{e}}\left(\frac{T_{0}}{R_{c}^{2}}-\frac{T_{1}{-T}_{0}}{R_{c}R_{h}}-\frac{T_{1}}{R_{h}^{2}}\right)$$

And

(7c) $$C_{2}=\sqrt{\frac{1}{R_{c}^{2}}+\frac{2}{{3R}_{c}R_{h}}+\frac{1}{R_{h}^{2}}+\frac{8}{{3R}_{t}}\left(\frac{1}{R_{c}}+\frac{1}{R_{h}}\right)+\frac{4}{3}\frac{N_{g}^{2}S_{g}^{2}}{R_{e}}\left(\frac{T_{0}}{R_{c}}+\frac{T_{1}}{R_{h}}\right)}$$

where $T_{1}$ and $T_{0}$ are the temperature at the hot end and cold end, respectively. The maximum output power can be obtained by matching the external electrical load:

(8a) $$P_{\mathrm{out}}=\frac{R_{e}}{{4N}_{g}^{2}S_{g}^{2}}{\left[\frac{1}{R_{c}}-\frac{1}{R_{h}}{+2C}_{2}\;\cos\left(\frac{1}{3}C_{2}\;\cos^{-1}\left(\frac{C_{1}}{C_{2}^{3}}\right)+\frac{4\pi }{3}\right)\right]}^{2}$$

and the output voltage

(8b) $$V_{\mathrm{out}}=\frac{R_{e}}{{2N}_{g}S_{g}}\left[\frac{1}{R_{c}}-\frac{1}{R_{h}}{+2C}_{2}\;\cos\left(\frac{1}{3}C_{2}\;\cos^{-1}\left(\frac{C_{1}}{C_{2}^{3}}\right)+\frac{4\pi }{3}\right)\right]$$

Note that the above derivation neglects the heat conduction loss of the metal layer(s) and insulation layer(s), as well as the convection loss to the isolation cavity around the thermocouples. Some 2D finite-element models have previously been proposed to investigate the convection loss of the thermal isolation cavity [9].

**Figure 2:**
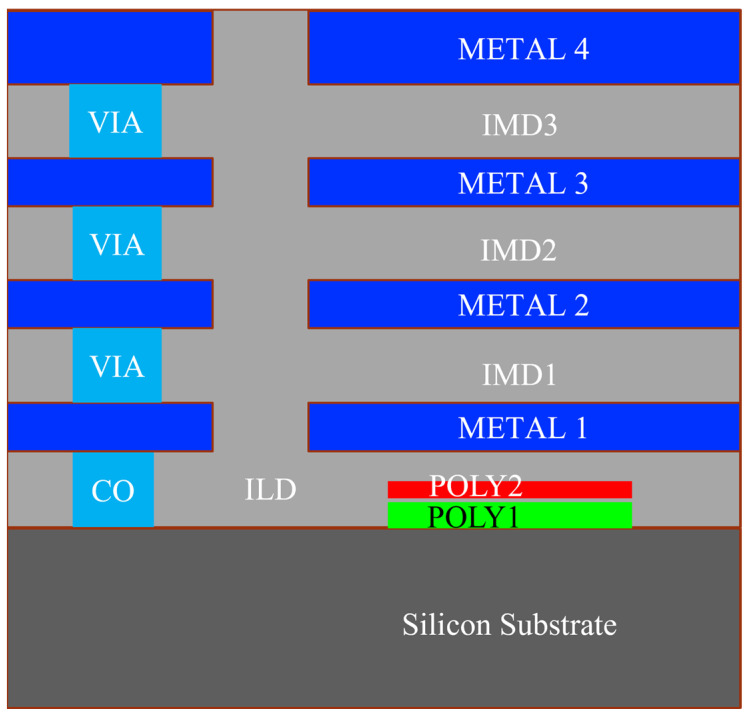
The CMOS process (TSMC 0.35 μm 2P4M TSMC) for implementing the TEG design with co-planar and with stacked thermocouples. The two polysilicon layers (POLY1 0.278 μm and POLY2 0.180 μm are for N- and P-thermoleg, respectively) and the four metal layers (METAL 1–4) are for circuit connection, hot/cold junctions, and etching masks. ILD and IMDs are interlayer dielectrics, VIA represents the copper-lined holes for circuitry, and CO is the metal contact.

**Figure 3:**
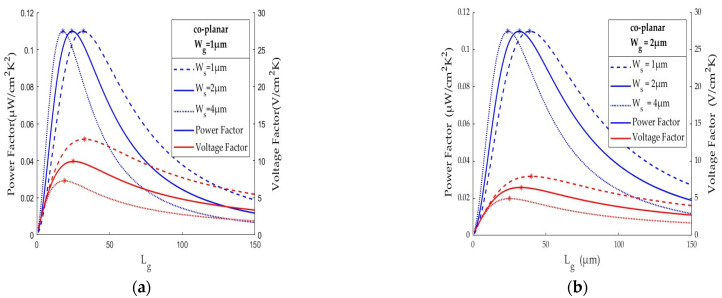
The performance of TEG with co-planar thermocouples at different area densities: (**a**) thermocouple width $W_{g}$ = 2 μm and (**b**) $W_{g}$ = 1 μm at width spacing $W_{s}$ = 1, 2, or 4 μm, where the maximum power factor and voltage factor are marked by *.

Consider a TEG with polysilicon thermocouples by the CMOS process (Taiwan Semiconductor Manufacturing Company (Hsinchu, Taiwan) TSMC 0.35 µm 2P4M) to evaluate the effect of the thermocouple area density on the power factor and voltage. The data of thermoelectric material properties are listed in Table 1 and the deposition layers are shown in Figure 2. For a semiconductor TEG operating in the body temperature range, $T_{1}$ = 308 K (35 °C) and $T_{0}$ = 298 K (25 °C), with thermocouple spacing of $L_{s}$ = 2 μm, the thermocouple width of $W_{g}$ = $W_{s}$ = 2 μm, and the hot/cold junction of $A_{h}$ = $A_{c}$ = 2 × 12 μm, Figure 3a shows the TEG performance simulation at different thermocouple lengths. The optimal length is obtained by having thermocouples of 33 × 2 µm ($L_{g}$ × $W_{g}$) in dimensions to achieve a power factor of 0.110 µW/cm^2^K^2^ and voltage factor of 6.453 V/cm^2^K. The area density of 2451 is about one-to-two orders higher than most of the other TEGs listed in [10]. The regions far from the maximum indicate thermal/electrical resistance mismatching. A thermocouple (of say 100 μm) with large thermal resistance leads to a small thermal flow. Conversely another (of say 20 μm) may result in a small temperature gradient across the hot/cold junctions. Both render a very low voltage and poor power. An optimal thermocouple dimension is therefore required to increase the temperature gradient while preventing Joule heating. The calculation of area density shall be based accordingly. Only with practical performance will the study of area density be meaningful.

## 3. Thermocouple Area Density

In the TEG design the thermocouple thickness, as listed in Table 1, is dictated by the thin-film deposition. The thermocouple width $W_{g}$ and the space $W_{s}$ between two adjacent thermocouples, as shown in Figure 1a, are determined by the “line width” (or process resolution) of CMOS process. The geometric parameters of a thermocouple to be determined are its length $L_{g}$ (often 20~150 μm), width $W_{g}$ (1~10 μm), and $W_{s}$ width spacing (1~10 μm). The aim is to apply silicon processing to increase the area density by reducing the thermocouple width, $W_{g}$, and the space, $W_{s}.$ It is apparent from Equation (2) that the voltage factor is a linear function of the thermocouple density, hence $W_{s}$, but the power factor remains the same as illustrated in Figure 3a because the higher the thermocouple density, the higher the number of thermocouples and thus the higher the thermocouple resistance.

The advantage of high thermocouple resistance is that it is in the same order of thermal resistance at the hot/cold interfaces, typically ~10 K/mW. Without a high area density, the mismatching thermal and electrical resistance can significantly hinder the heat flow from the hot/cold side to the hot/cold junctions. The impact can be observed from the inadequate thermocouple size of a short thermocouple of 20 × 2 µm ($L_{g}$ × $W_{g})$ with a power factor of 0.040 W/cm^2^K^2^ and a voltage factor of 3.656 V/cm^2^K in Figure 3a, where the thermal resistance is only 1.216 K/W. This is about the same as that (1.555 K/W) of the long thermocouple of 120 × 40 µm, found by the authors in [21]. Such thermal resistance leads to a small temperature gradient across the thermocouples and therefore a poor power factor 0.00363 μW/cm^2^K^2^ and low voltage factor 0.746 V/cm^2^K in [21]. Both numbers are 1~2 orders lower than those of the optimal thermocouple size of 33 × 2 μm at an area density of 2451.

Figure 3a also shows the power factor and voltage factor of a TEG with co-planar thermocouples of $W_{g}$ = 2 μm and $W_{s}$ = 1 μm at different $L_{g}$ values. The optimal length is increased to 40 μm with $\phi_{P}$ = 0.110 μW/cm^2^K^2^ and $\phi_{V}$ = 7.497 V/cm^2^K. The power factor remains unchanged but the voltage factor has been increased by 23%. Evidently, the area density is increased from 2451 to 3049 thermocouples per mm^2^ by the smaller width spacing. Compared with the TEG design used more than a decade ago, which was limited by the processing resolution at $W_{s}$ = 4 μm [9], Figure 3a also shows that the optimal length is 25 μm with $\phi_{P}$ = 0.110 μW/cm^2^K^2^ and $\phi_{V}$ = 4.955 V/cm^2^K, with the area density of 1923 thermocouples per mm^2^. These results confirm that reducing the width spacing, $W_{s}$, is beneficial to the high area density, and hence high performance. In addition, the high thermal resistance matching that at the TEG interface(s) is also beneficial to effective energy harvesting. The power factor and voltage factor of a TEG with $W_{g}$ = 2 μm and 4 μm have been validated in the authors’ recent works summarized in [10].

The area density can also be increased by a smaller thermocouple width $W_{g}$. Figure 3b illustrates the performance simulation of a TEG with co-planar thermocouples of width $W_{g}$ = 1 μm at $W_{s}$ = 1, 2, or 4 μm and different thermocouple lengths. Again, the optimal performance is dependent upon the thermocouple size. At $W_{s}$ = 1 μm, the optimal thermocouple is 33 × 1 μm ($L_{g}$ × $W_{g}$) at an area density of 4902 thermocouples per mm^2^ to reach $\phi_{P}$ = 0.110 μW/cm^2^K^2^ and $\phi_{V}$ = 12.906 V/cm^2^K. For a 5 × 5 mm^2^ TEG chip, there are 122,500 thermocouples of 33 × 1 μm at a width spacing of $W_{s}$ = 1 μm. It can deliver about 3 μW and over 3 V when operating at a 10 °C temperature difference. The area density is about 62% higher than that of the TEG at $W_{g}$ = 2 μm, shown in Figure 3a, and so is the voltage factor. Similarly, at $W_{s}$ = 2 μm, the optimal size is 25 × 1 μm at an area density of 3846 thermocouples per mm^2^ to $\phi_{P}$ = 0.110 μV/cm^2^K^2^ and $\phi_{V}$ = 9.911 V/cm^2^K. Reducing $W_{g}$ and/or $W_{s}$ for a high area density are both effective to improve TEG performance. The thermocouple size is critical to its output power and voltage in TEG operation. A thermocouple of geometry matching its thermal/electrical resistance is key to optimal TEG performance, as demonstrated in Figure 3a,b. Table 2 summarizes the effect of thermocouple geometric parameters, and hence the area density, on TEG performance. A high area density with a thermocouple size matching its thermal and electrical resistance is key to improving TEG performance.

## 4. Stacked Thermocouples

**Figure 4:**
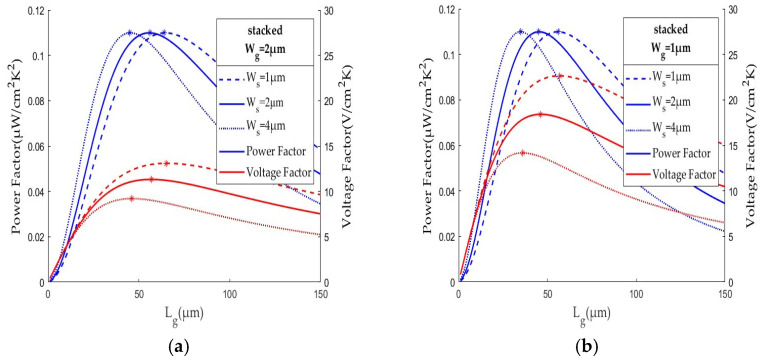
The performance of TEG with stacked thermocouples of (**a**) width $W_{g}$ = 2 μm and (**b**) $W_{g}$ = 1 μm at different width spaces ($W_{g}$ = 1, 2, or 4 μm), where the maximum power factor and voltage factor are marked by *.

The stacked thermocouple design, as illustrated in Figure 1b, has been shown to increase performance by a high area density [14]. When placing the P-thermoleg on top of the N-thermoleg, the performance of a TEG with stacked polysilicon thermocouples has been analyzed and validated [11] to be better than that of a TEG with co-planar thermocouples. Figure 4a shows the performance of a TEG with a stacked thermocouple design at $W_{g}=W_{s}$ = 2 μm. At the optimal length, $L_{g}^{*}$ = 57 μm, the power factor is 0.110 μW/cm^2^K^2^ and the voltage factor is 11.319 V/cm^2^K. Both factors are higher than the TEGs with co-planar thermocouples of the same $W_{g}$ and $W_{s}$. The area density increase from 2451 to 4310 thermocouples per mm^2^ is shown to achieve a higher voltage output. At a smaller width spacing of $W_{s}$ = 1 μm, the area density is 5050 thermocouples per mm^2^ and the optimal length is 65 μm with $\phi_{P}$ = 0.110 μW/cm^2^K^2^ and $\phi_{V}$ = 13.100 V/cm^2^K. Similarly, at $W_{s}$ = 4 μm, the area density is 3546 thermocouples per mm^2^ with $\phi_{P}$ = 0.110 μW/cm^2^K^2^ and $\phi_{V}$ = 9.206 V/cm^2^K. The high area density by the stacked thermocouple design is beneficial to high TEG performance. Figure 4b also shows the performance of TEG width with stacked thermocouples of $W_{g}$ = 1 μm at $W_{s}$ = 1, 2, and 4 μm. The smaller the $W_{s}$, the higher the area density and the higher the voltage factor. Table 3 summarizes the effect of area density on the performance of a TEG with stacked thermocouples. The area density of the TEG with stacked thermocouples is higher than that of the TEG with co-planar thermocouples. The former achieves higher TEG performance. For a 5 × 5 mm^2^ TEG chip, there are 215,520 stacked thermocouples of 57 × 1 μm ($L_{g}$ × $W_{g}$) at $W_{s}$ = 1 μm width spacing. It can generate about 3 μW and over 3 V when operating at a 10 °C temperature difference.

This study demonstrates that the area density is a good indicator of TEG performance. A high area density can raise the voltage factor. Compared with the studies reported in the literature after 2021, the TEG studied by the authors in [22] has 6400 polysilicon thermocouples of size 75 × 20 μm ($L_{g}$ × $W_{g}$) with a fill factor of 19.6% (equivalent area density of 131 thermocouples per mm^2^) for a 0.00634 μW/cm^2^K^2^ power factor and a 0.316 V/cm^2^K voltage factor. Its area density, power factor, and voltage factor were about 1~2 orders smaller than this study.

The TEG studied by the authors in [6] has 54 polysilicon thermocouples of size 180 × 26 μm with a fill factor of 26.3% (equivalent area density of 108 thermocouples per mm^2^) for a 0.031 μW/cm^2^K^2^ power factor and a 0.893 V/cm^2^K voltage factor. These studies with a poor power factor and impractical voltage factor typify the need to boost TEG performance with high area density. A high voltage factor is necessary to acquire 1.5 or 3 V in applications and to match the impedance of the DC–DC converter [23].

The challenge of wearable TEGs is to deliver sufficient voltage and power levels in electronics at a typical temperature difference, for example 10 °C. A TEG with a mm^2^ footprint with high area density over 10^3^ of the thermocouples for on-chip integration has been shown to generate higher than a 3 V voltage with a µA current to drive silicon transistors. A high area density of thermocouples is therefore critical to TEG applications. With the stacked thermocouple, a 5 × 5 mm^2^ TEG chip can deliver 3 μW and over 3 V with a 10 °C temperature difference. Recent studies to increase the figure of merit reported a TEG with silicon nanowire thermocouples generating a 0.0000213 μW/cm^2^K^2^ power factor with a 0.0045 V open-circuit voltage [5], and another TEG with silicon germanium nanowire thermocouples generating a 0.000509 μW/cm^2^K^2^ power factor with a 0.0138 V open-circuit voltage [24]. These further indicate that a high area density of thermocouples is much more effective to improve TEG performance than the thermocouple materials with a high figure of merit. The standard CMOS process is clearly advantageous to TEG design in delivering a 10^−1^ µW/cm^2^K^2^ power factor and ~10^1^ V/cm^2^K voltage factor. The device (geometry, size, connection, current, and heat flow) and the implementation to interfaces (thermocouple materials, electrodes, and insulating substrates) are the key in TEG design. Among these design parameters, a high thermocouple area density is the way towards achieving wearable TEGs.

## 5. Conclusions

The high area density of thermocouples in TEGs can be implemented by a standard CMOS process, where batch production, device scalability, and production cost-effectiveness further boost TEG performance. Semiconductor TEG design in a hybrid configuration is preferable because of the sufficient thermocouple length to harness the temperature gradient over the hot/cold junction. The heat flow from the hot side is confined within the in-plane thermocouples for better thermoelectric conversion. A sufficient temperature gradient within a thermocouple is necessary by matching its electrical/thermal resistance. The key to high TEG performance is to increase the area density of thermocouples at about 2000~8000 per mm^2^. In TEG operation, a boost DC–DC converter is needed to match the input voltage to the wearable device(s). The converter efficiency by matching the impedance of the TEG to maximize the output power is needed to effective TEG operation. Having a high area density for a high voltage factor is therefore critical to the TEG design.

Many previous semiconductor TEGs with a poor output power (~nW/cm^2^K^2^) and low operating voltage (~mV/cm^2^K) used by the authors in [10] were the result of a low thermocouple area density and an “incorrect” thermocouple size. The prerequisite of a high area density is to determine the thermocouple dimension so as to match its thermal/electrical resistance. It has been shown that the area density, defined by the number of thermocouples per ${\mathrm{mm}}^{2}$, is a better index than the fill factor in evaluating performance. By packing more thermocouples of optimal size, one can have a higher thermocouple thermal resistance and voltage factor. Both are critical to impedance matching and voltage regulation in TEG operation. An optimal thermocouple dimension is required to increase thermal flow while preventing Joule heating.

TEGs with semiconductor thermocouples by a standard CMOS process (TSMC 0.35 μm 2P4M) have been shown by simulation to achieve a ~10^−1^ μW/cm^2^K^2^ power factor and ~10^1^ V/cm^2^K voltage factor. The high area density of thermocouples can engineer thermal/electrical impedances with the control unmatched by any other processes. For TEGs with co-planar thermocouples, one can narrow the thermocouple width $W_{g}$ and width space $W_{s}$ for a high area density. Analysis shows that a CMOS TEG with a 33 × 1 μm thermocouple at a width space of $W_{s}$ = 1 μm can achieve an area density of 4902, along with a 0.110 μW/cm^2^K^2^ power factor and a 12.906 V/cm^2^K voltage factor. The performance can be further improved by the stacked thermocouple design at a size of 57 × 1 μm for an area density of 8621, with a 0.110 μW/cm^2^K^2^ power factor, and a 22.638 V/cm^2^K voltage factor. In the mass production of a CMOS TEG, an 8” wafer could produce more than 1000 5 × 5 mm^2^ TEG dies, so the challenge is not in the technical aspects, but in the economical projection.

## Figures and Tables

**Table 1:** The thermoelectric and geometric properties for calculating TEG area density and performance (TSMC 0.35 μm 2P4M CMOS process).

Symbol	Description	Value
	Material Property	
S_p_ S_n_ ρ_p_ ρ_n_ k_p_ k_n_ k_c_ k_i_	Seebeck coefficient of P-thermolegs (µV/K) Seebeck coefficient of N-thermolegs (µV/K) Electrical resistivity of P-thermolegs (mΩ-cm) Electrical resistivity of N-thermolegs (mΩ-cm) Thermal conductivity of P-thermolegs (W/mK) Thermal conductivity of N-thermolegs (W/mK) Thermal conductivity of Si (W/mK) Thermal conductivity of SiO_2_ (W/mK)	120.22 15.8 1.015 0.105 31.2 31.5 168 1.1
	Geometry Parameter	
t_p_ t_n_ t_c_	Thickness of P-thermolegs (µm) Thickness of N-thermolegs (µm) Thickness of Si substrate (µm)	0.275 0.180 650

**Table 2:** Performance of TEG with co-planar thermocouples of width $W_{g}$ = 1 and 2 μm at different width spacings, $W_{s}$, where ${L_{g}}^{*}$ is the optimal thermocouple length, $A_{d}$ is the area density, and $N_{g}$ is the number of thermocouples in 5 × 5 mm^2^ chip.

Symbol	Co-planar $W_{g}$ = 1 μm		
$W_{s}$	1	2	4
$\phi_{P}$	0.110	0.110	0.110
$\phi_{V}$	12.906	9.911	7.293
${L_{g}}^{*}$	33	25	19
$A_{d}$	4902	3846	2778
$N_{g}$	122,550	96,150	69,440
	Co-planar $W_{g}$ = 2 μm		
$W_{s}$	1	2	4
$\phi_{P}$	0.110	0.110	0.110
$\phi_{V}$	12.906	9.911	7.293
${L_{g}}^{*}$	33	25	19
$A_{d}$	4902	3846	2778
$N_{g}$	122,550	96,150	69,440

**Table 3:** Performance of TEG with stacked thermocouples with width $W_{g}$ = 1 and 2 μm at different width spacings, $W_{s}$, where ${L_{g}}^{*}$ is the optimal thermocouple length, $A_{d}$ is the area density, and $N_{g}$ is the number of thermocouples in 5 × 5 mm^2^ chip.

Symbol	Stacked $W_{g}$ = 1 μm		
$W_{s}$	1	2	4
$\phi_{P}$	0.110	0.110	0.110
$\phi_{V}$	22.638	18.412	14.174
${L_{g}}^{*}$	57	46	36
$A_{d}$	8621	7092	5406
$N_{g}$	215,520	117,300	135,140
	Stacked $W_{g}$ = 2 μm		
$W_{s}$	1	2	4
$\phi_{P}$	0.110	0.110	0.110
$\phi_{V}$	13.110	11.319	9.206
${L_{g}}^{*}$	65	57	46
$A_{d}$	5050	4310	3546
$N_{g}$	126,260	107,760	88,650

## Data Availability

The data presented in this study are available on request from the corresponding author.
